# Bio-distribution of Carbon Nanoparticles Studied by Photoacoustic Measurements

**DOI:** 10.1186/s11671-022-03768-3

**Published:** 2022-12-23

**Authors:** Kateryna Dubyk, Tatiana Borisova, Konstantin Paliienko, Natalia Krisanova, Mykola Isaiev, Sergei Alekseev, Valeriy Skryshevsky, Vladimir Lysenko, Alain Geloen

**Affiliations:** 1 Taras Shevchenko National University of Kyiv, 64/13, Volodymyrska Street, Kyiv, 01601 Ukraine; 2 Corporation Science Park, Taras Shevchenko University of Kyiv, 60, Volodymyrska Street, Kyiv, 01033 Ukraine; 3 Palladin Institute of Biochemistry, National Academy of Sciences of Ukraine, 9 Leontovicha Street, Kiev, 01054 Ukraine; 4 Université de Lorraine, CNRS, LEMTA, F-54000 Nancy, France; 5 Light Matter Institute, UMR-5306, Claude Bernard University of Lyon/CNRS, Université de Lyon, 69622 Villeurbanne Cedex, France; 6 UMR Ecologie Microbienne Lyon (LEM), CNRS 5557, INRAE 1418, Claude Bernard University of Lyon, VetAgro Sup, Research Team “Bacterial Opportunistic Pathogens and Environment” (BPOE), University of Lyon, 69622 Villeurbanne, France

**Keywords:** Photoacoustics, Contrast agents for photoacoustic tomography, Carbon nanodots, Carbon flurooxide nanoparticles, Intravenous injection, Autological biocorona, Rats

## Abstract

Carbon-based nanomaterials are promising for a wide range of biomedical applications, i.e. drug delivery, therapy, and imaging including photoacoustic tomography, where they can serve as contrast agents, biocompatibility and biodistribution of which should be assessed before clinical setting. In this paper, localization of carbon flurooxide nanoparticles, carbon nanodots from *β*-alanine, carbon nanodots from urea and citric acid and glucose-ethylenediamine nanoparticles (NPs) in organs of Wistar rats were studied by photoacoustic measurements after 24 h of their intravenous injection. 16 ns light pulse from a Q-switched Nd:YAG laser with 1064 nm wavelength was used as an excitation source. The laser-induced photoacoustic signals were recorded with a ring piezoelectric detector. Light absorption by carbon NPs resulted in noticeable enhancement of the photoacoustic amplitude in the tissues where the NPs were accumulated. The NPs were preferably accumulated in liver, kidneys and spleen, and to a lesser extent in heart and gastrocnemius muscles. Together with remarkable fluorescent properties of the studied carbon nanomaterials, their photoacoustic responses allow their application for bi-modal fluorescence-photoacoustic bio-imaging.

## Introduction

Photoacoustic tomography is a non-invasive and non-ionizing imaging technique combining high optical specificity and sensitivity with high resolution and penetration depth of ultrasound [1]. It remains one of the most perspective techniques for theranostic applications in humans [2]. It is a hybrid imaging modality, in which photons are absorbed and converted to heat, and the transient thermoelastic expansion of the heated biological tissue leads to emission of acoustic waves [3]. The photoacoustic waves are wide-band ultrasonic oscillations and in order to form images, they are detected by a high-frequency focused ultrasonic transducer [3]. Non-transparent samples can be subjected to photoacoustic detection that is exploited for characterization of biologic tissues and objects, where optical spectroscopic methods cannot be applied [2]. Optical wavelengths in the near infrared (700–1500 nm) are applied in biology and biomedicine because the tissues are quite transparent in this spectral range and penetration depth of the near-infrared light can reach up to several centimeters.

Photoacoustic signal can be considerably enhanced by means of special contrast agents. A potential contrast material for the photoacoustic imaging should possess both features, namely: (i) sufficient photon absorbance and (ii) high conversion efficiency from light to heat [3]. A wide variety of exogenous contrast agents with superior absorption coefficients in the near-infrared spectral region have been used for photoacoustic imaging, for example: gold-, silver-, copper agents [4–6], porphyrin, lipid and polypyrrole agents [7, 8], semi-conducting polymer agents [9]. Carbon nanomaterials appear also as perfect candidates for the photoacoustic imaging [3, 10, 11].

The nonradiative conversion of light energy into sound energy (used for photoacoustic imaging), as well as into heat (used for photothermal therapy), has been actively explored for diagnostics and treatment of cancer, which is currently a major cause of morbidity and mortality worldwide [12]. Photothermal transduction agents harvest the energy from light and convert it into heat thereby increasing the temperature of the surrounding cells and tissues and triggering the death of cancer cells [13, 14]. Photothermal therapy is thus an effective and noninvasive therapy capable to eliminate many types of cancers [15].

Biodistribution and toxicity of nanoparticles are key issues for their use as contrast agents in nanomedicine [16]. It depends on a wide variety of nanoparticle parameters, including size, shape, surface charge, core composition and ability to form protein/lipid biocorona at their surface during their interaction with physiological fluids [17]. Photoacoustic imaging, as a hybrid imaging modality, can provide sufficient optical contrast and high spatial resolution, making it a powerful tool for in vivo vascular imaging [18], blood lymphatic vessels [19], in nanodentistry [20] and in many others medical applications. Various methods for analysis of nanoparticle biodistribution, for example: histology and diverse microscopies require labor-intensive sample preparation. Thus, there is a need to develop unlabored approach of quantitative assessment of nanoparticle biodistribution [16].

Electrochemical synthesis of luminescent carbon flurooxide nanoparticles was previously described [21]. This kind of carbon-based nanoparticles has an organic-like nature. Their chemical composition can be described by a generalized formula: C_X_H_Y_O_Z_F_T_. The 1–10 nm large nanoparticles can be easily dispersed in water. The carbon flurooxide nanoparticles possess an ability to penetrate inside the cells, and they were used as fluorescent cellular labels and sonosensitizers for theranostic application [22].

Carbon nanodots possess a strongly defected composition with aromatic and aliphatic regions with graphene, graphene oxide and diamond zones [23]. The water-suspended carbon nanodots are characterized by inorganic carbon cores composed of *sp*^2^- hybridized graphene islands with diamond-like *sp*^3^-hybridized inclusions. The shells of carbon nanodots are represented by amorphous carbon and oxygen-containing polar groups (carbonyls, carboxyls and hydroxyls). The nitrogen-containing groups in –NH_2_ state, and as aromatic amines included into pyrrole rings can be also presented at the carbon nanodots surface [24, 25]. The size of uncoated carbon nanodots varied from 1 to 6 nm [23, 26, 27]. Previously, strongly fluorescent, non-blinking and emission-color-tuning carbon nanodots were synthesized and well characterized regarding their surface and fluorescent properties, their neurotoxicity and membrane-activity [23, 28]. It was revealed that carbon dots synthesized from *β*-alanine with use of microwave heating were more active regarding modulation of excitatory and inhibitory neurotransmitter transport in brain nerve terminals as compared to those synthesized from thiourea and citric acid [23, 28].

**Fig. 1:**
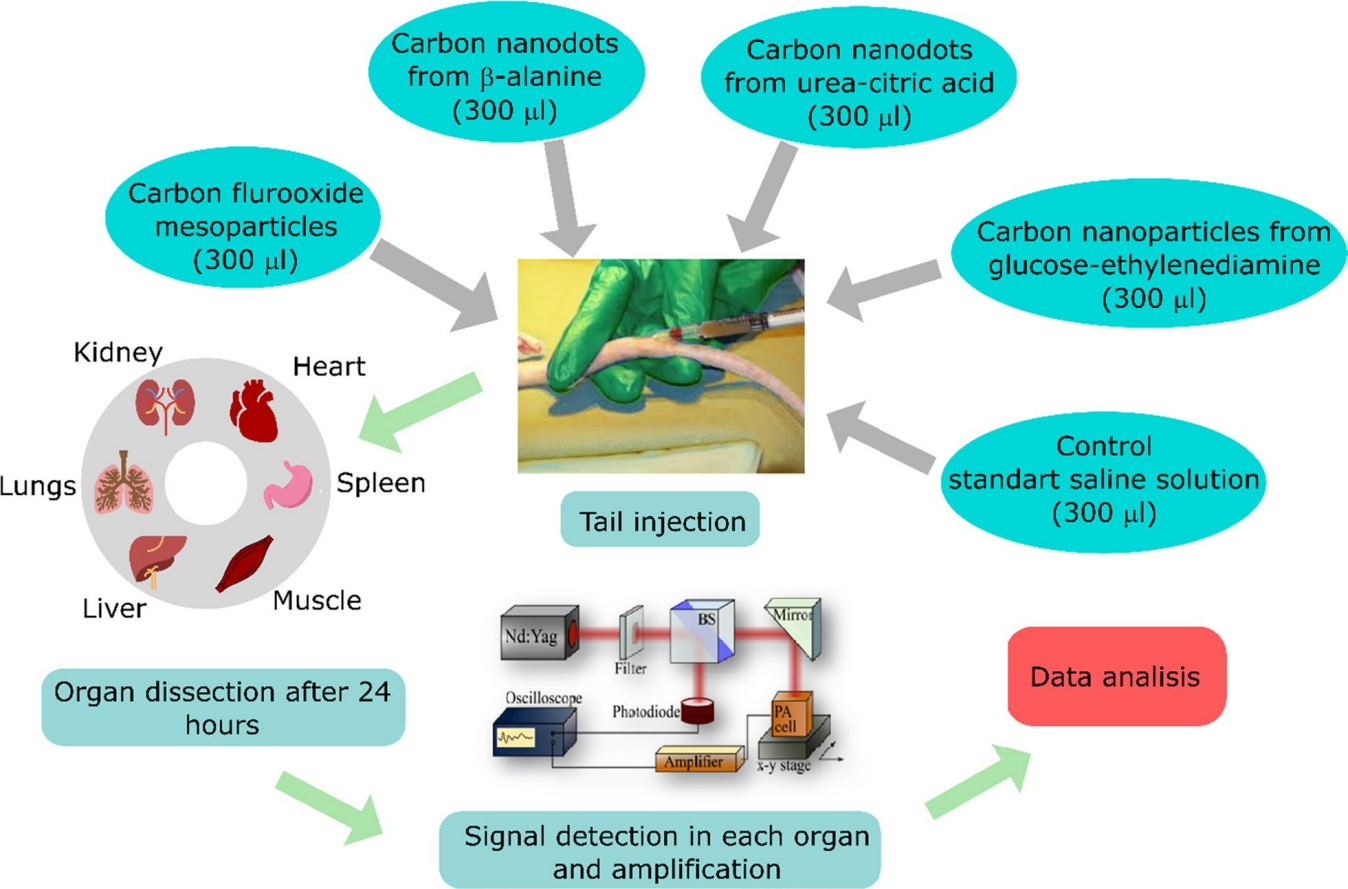
Experimental design roadmap

The main aim of this work was photoacoustic diagnostics of bio-distribution of carbon nanomaterials in organs (heart, liver, spleen, kidneys, muscle and lungs) after 24 h from intravenous injection of the nanoparticles to the rat’ tail vein. Four different types of the nanomaterials, such as: carbon flurooxide nanoparticles, carbon nanodots synthesized from *β*-alanine and urea-citric acid using microwave heating, and glucose-ethylenediamine nanoparticles prepared by hydrothermal synthesis were used in this study. The design of the experiments is shown in Fig. 1. Before the injection, all nanoparticles were mixed with the blood plasma, thereby being coated with autological biocorona from plasma proteins.

## Materials and Methods

### Materials

Crystalline citric acid (99% purity grade, Sigma-Aldrich), crystalline urea (98% purity grade, Sigma-Aldrich), *β*-alanine (95% purity grade, Reanal), salts NaCl, KCl, MgCl_2_, NaH_2_PO_4_, HEPES (pH 7.4) were of analytical grade and were purchased from Sigma-Aldrich.

### Synthesis of Carbon Nanomaterials

#### Carbon Flurooxide Nanoparticles

The carbon flurooxide nanoparticles were fabricated via electrochemical etching of low-resistivity grade (0.7 mΩ cm) *n*-type 3C-SiC bulk polycrystalline substrate, according to previously reported protocols [29, 30]. In brief, the anodization was performed in HF (48%)-ethanol (1:1, v/v) mixture for 3 h at 25 mA/cm^2^. After the etching, the wafer was gently washed several times with deionized water and then naturally dried in ambient conditions. The brown layer, formed onto the substrate after anodization, was collected by mechanical scratching giving a powder, which was a porous SiC (approx. 5–8 wt%) and the carbon flurooxide nanoparticles (92–95 wt%) mixture. To prepare the solution for the experiment, the powder (1 mg/mL) was dispersed directly in a buffer solution under sonication. The liquid was centrifuged to remove the SiC admixture, giving clear brown solution.

#### Carbon Nanodots from β-alanine

Carbon nanodots from *β*-alanine were synthesized according to the previously reported method [31] with some modifications. Batch 1 g of *β*-alanine was heated in a tube in electric heating oven at 160 °C for 20 min. Then, 4 mL of bidistilled water was added to the tube, mixed on vortex shaker. Concentration of carbon dots was determined by gravimetric method. Stock solution of nanoparticles was dissolved in distilled water.

#### Carbon Nanodots from Urea and Citric Acid

Carbon nanoparticles from citric acid (CAc) and urea were prepared using a high-temperature treatment [31, 32]. In particular, citric acid (500 mg) was mixed with urea (500 mg). This dry mixture was heated in high-temperature electric oven at 160 °C during 5 min. Then, 4 mL of bidistilled water was added, and mixed at vortex shaker. To concentrate, the suspension was boiled until the volume was reduced two times, and then the mixture was sedimented in a microcentrifuge at 4300 g for 5 min, and then the supernatant was collected. Concentration of carbon nanodots from CAc and urea was measured analogically as described in the previous subsection for those from *β*-alanine.

#### Glucose-Ethylenediamine Nanoparticles

The glucose-based carbon nanoparticles were synthesized according to the procedure previously described in [33]. Glucose monohydrate (0.495 g, 2.5 mmol) was dissolved in 5 mL of water, then ethylenediamine (170 μL, 2.5 mmol) was added. The mixture was heated at 180 °C for 3 h in an autoclave. Resulted solution was evaporated at 60 °C in a rotary evaporator and additionally dried at 60 °C under 10^−3^ bar vacuum. The solid was dissolved in water (1 mg/mL) under sonication giving clear brown solution; negligibly small precipitate was removed by the centrifugation.

### Nanoparticle Size and Zeta Potential Measurements

Sizes and zeta potentials of the synthesized nanoparticles were determined by dynamic light scattering method using Malvern Zetasizer Nano ZS (Malvern Panalytical Ltd) instrument at 173° backscatter geometry for 0.5 mg/mL solutions of the nanoparticles, the refractive index of the nanoparticles was taken as 1.5.

**Fig. 2:**
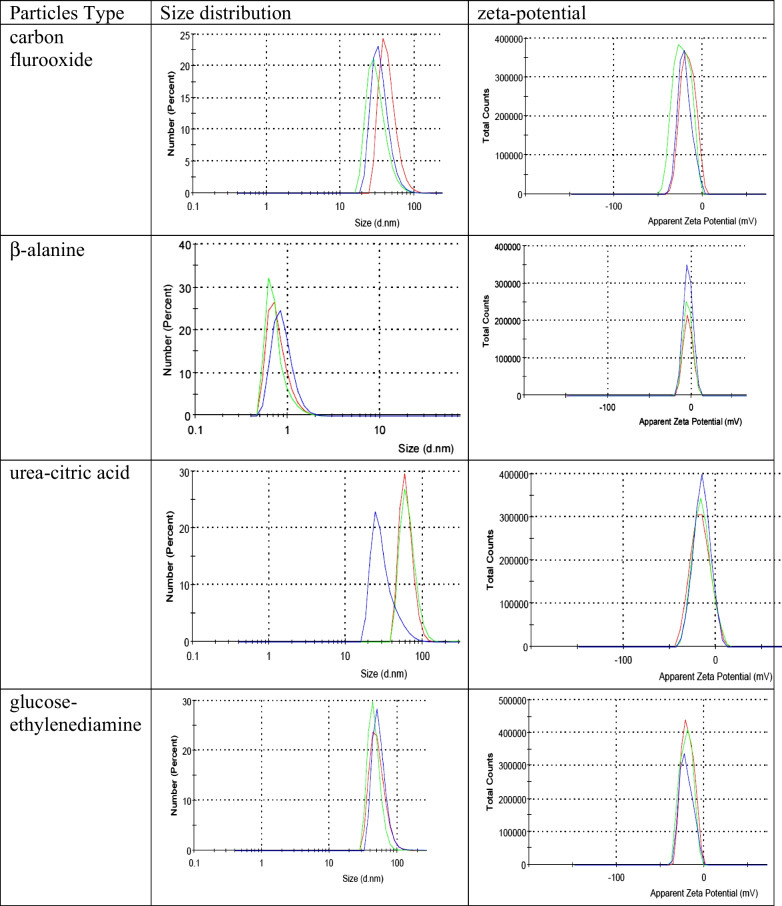
Size distribution and zeta-potential of nanoparticle samples were measured for carbon flurooxide nanoparticles, carbon nanodots from *β*-alanine, carbon nanodots from urea-citric acid, and glucose-ethylenediamine nanoparticles. Each measurement was carried out during 1 min. Red line: first measurement, green line: second, blue line: third

The largest carbon flurooxide nanoparticle population consisted of uniform 45 ± 15 nm particles with zeta-potential ζ = − 16.4 mV; carbon nanodots from *β*-alanine consisted of 99% of subnanometer particles with zeta-potential ζ = − 3.6 mV; carbon nanodots from urea-citric acid consisted of the largest NPs with sizes near 62 ± 13 nm and zeta-potential ζ = − 16.8 mV; the largest glucose-ethylenediamine nanoparticle sizes were about 52 ± 14 nm with zeta-potential ζ = − 18.6 mV (Fig. 2).

### Ethics

Wistar male rats with body weight near 150 g were kept in the animal facilities of the Palladin Institute of Biochemistry, National Academy of Sciences of Ukraine, housed in a quiet, temperature-controlled (22–23 °C) vivarium and were provided with water and dry food pellets *ad libitum*. All procedures were conducted according to the Declaration of Helsinki (“Scientific Requirements and Research Protocols” and “Research Ethics Committees”). Experimental protocols were approved by the Animal Care and Use Committee of the Institute (Protocol from 3/09-2018). Before removing the organs, rats were anesthetized. The total number of animals used in the study was 15, namely 3 control animals, and the rest—three animals for each type of nanoparticles.

### Intravenous Injection Procedure

A standard saline solution contained 126.0 mM NaCl, 4.0 mM KCl, l.4 mM MgCl_2_, 1.0 mM NaH_2_PO_4_, 20.0 mM HEPES (pH 7.4) was used for dilution of carbon nanomaterials before intravenous injection. Stock solution of carbon flurooxide nanoparticles was prepared using the standard saline solution, i.e. 10 mg of the nanoparticles were mixed with 1 ml of the solution using vortex shaker within 5 min. Then pH-value was adjusted up to pH 7.0 using 0.01M NaOH and mixed again for 5 min.

Rats were anaesthetized with chloroform for 2 min before being injected. Injections of carbon nanomaterials diluted in accordance to protocols presented in Table 1 were made into the tail vein of rats. Heparin 5 MO per 300 µl was added to injection solution. Control rats were injected with the standard saline solution. All nanoparticles were mixed with the blood plasma in plastic syringe within injection procedure, thereby being coated with autological biocorona of the plasma proteins [34]. Rats’ body weights were determined before injection and 24 h post nanomaterial administration using electronic scales.

**Table 1:** Injection protocols

Injection material	Injected volume, µl	Concentration of nanoparticles per body weight, µg/g
Control (standard saline solution)	300	0
Carbon flurooxide nanoparticles	300	18.19 ± 3.88
Carbon nanodots (*β*-alanine)	300	9.44 ± 0.77
Carbon nanodots (urea + CAc)	300	30.20 ± 0.20
Glucose-ethylenediamine nanoparticles	300	2.99 ± 0.46

### Tissue Sampling

Rats’ organs were collected for photoacoustic analysis after 24 h from intravenous administration of carbon nanomaterials. Rats were anesthetized and killed by rapid decapitation and after that different organs were dissected and the samples were frozen.

### Photoacoustic Signal Generation

**Fig. 3:**
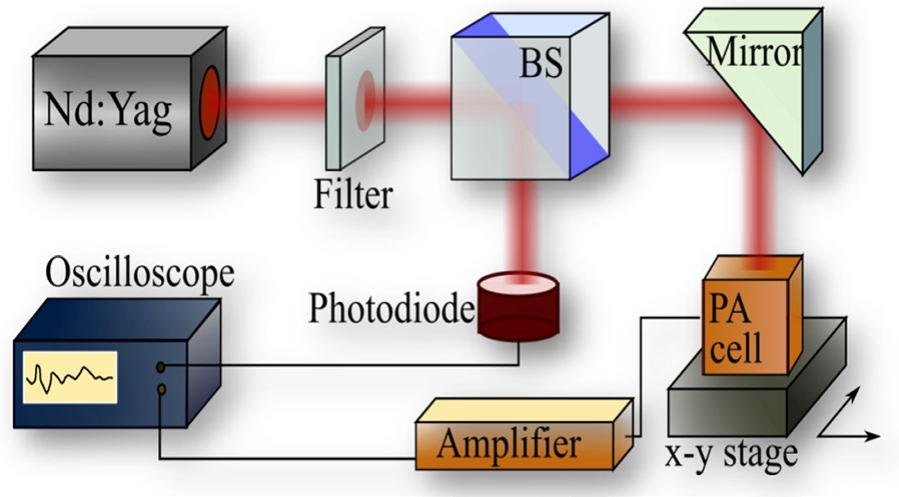
Scheme of the photoacoustic experimental setup

16 ns pulse near-infrared light radiation from a Q-switched Nd:YAG laser (with 1064 nm wavelength) was used as an excitation source. A simplified scheme of the photoacoustic experimental set-up is shown in Fig. 3. The pulse energy was reduced by a filter to avoid any tissue damage.

Additionally, the intensity of the laser beam was controlled with a photodiode. The beam was directed toward a photoacoustic probe put in contact with studied tissue sample through a transmission gel. Generated photoacoustic signals were recorded with a piezoelectric ring, amplified and visualized on a digital oscilloscope. The final oscillogram was averaged among 128 pulses.

The generation of a photoacoustic response can be described with the following three steps: (i) absorption of the radiation by a studied object, (ii) conversion of the absorbed radiation into the heat and appearing of the temperature rise in the heated region, and (iii) thermoelastic expansion resulting in the emission of acoustic waves.

Following a short laser pulse excitation, the local fractional volume expansion d*V*/*V* of the heated tissue at position $\vec{r}$ can be expressed [35]:

1 $$\frac{{{\Delta }V}}{V} = - k{\Delta }p\left( {\vec{r}} \right) + \beta {\Delta }T\left( {\vec{r}} \right)$$

where *k* is the isothermal compressibility, *β* is the thermal coefficient of volume expansion, and $\Delta p(\vec{r})$ and $\Delta T(\vec{r})$, are changes in pressure and temperature, respectively. The isothermal compressibility was equal to ∼ 5 × 10^−10^ Pa^−1^ for water or soft tissue, the thermal coefficient of volume expansion was equal to ∼ 4 × 10^−4^ K^−1^ for muscle [36].

For effective PA signal generation, the laser pulse duration should be within several nanoseconds, which is less than both thermal and acoustic confinement times:

2 $$\tau < \frac{{l_{c} }}{{v_{s} }} < \frac{{l_{c}^{2} }}{{4\alpha_{{{\text{th}}}} }}$$

where *l*_c_ is the characteristic length of heat heterogeneity (the desired spatial resolution), and *α*_th_ is the thermal diffusivity (∼ 0.1 mm^2^/s for tissue) [35].

For a short laser pulse, the fractional volume expansion is negligible, band the local pressure rise *p* immediately after the laser excitation [37] can be derived from Eq. (1) as:

3 $${\Delta }p\left( {\vec{r}} \right) = \frac{{\beta {\Delta }T\left( {\vec{r}} \right)}}{k}$$

The temperature rise can be further expressed as a function of optical absorption:

4 $$T = \frac{{A_{e} }}{{\rho C_{V} }}$$

here *A*_*e*_ is the specific or volumetric optical absorption, *ρ* is the mass density (∼ 1000 kg/m^3^ for water and soft tissue), *C*_*V*_ is the specific heat capacity at constant volume (∼ 4000 J/(kg K)for muscle).

The photoacoustic pressure can be written as:

5 $$p = \frac{\beta }{{k\rho C_{V} }}A_{e} = \Gamma A_{e}$$

here *Г* is the Grueneisen parameter.

Thus, variation of the optical absorption of the media leads to the variation of the registered photoacoustic response magnitude.

## Results

Body weights of animals were determined before and 24 h after the injection procedure. The relative decrease in the rats’ body weights was 7.8 ± 1.3 % in the control group, 6.8 ± 0.1 % after injection of carbon flurooxide nanoparticles, 4.2 ± 3.4 % after injection of carbon nanodots from *β*-alanine, 1.6 ± 1.3 % after injection of carbon nanodots from urea-citric acid, and 7.4 ± 2.9 % after injection of glucose-ethylenediamine nanoparticles.

**Fig. 4:**
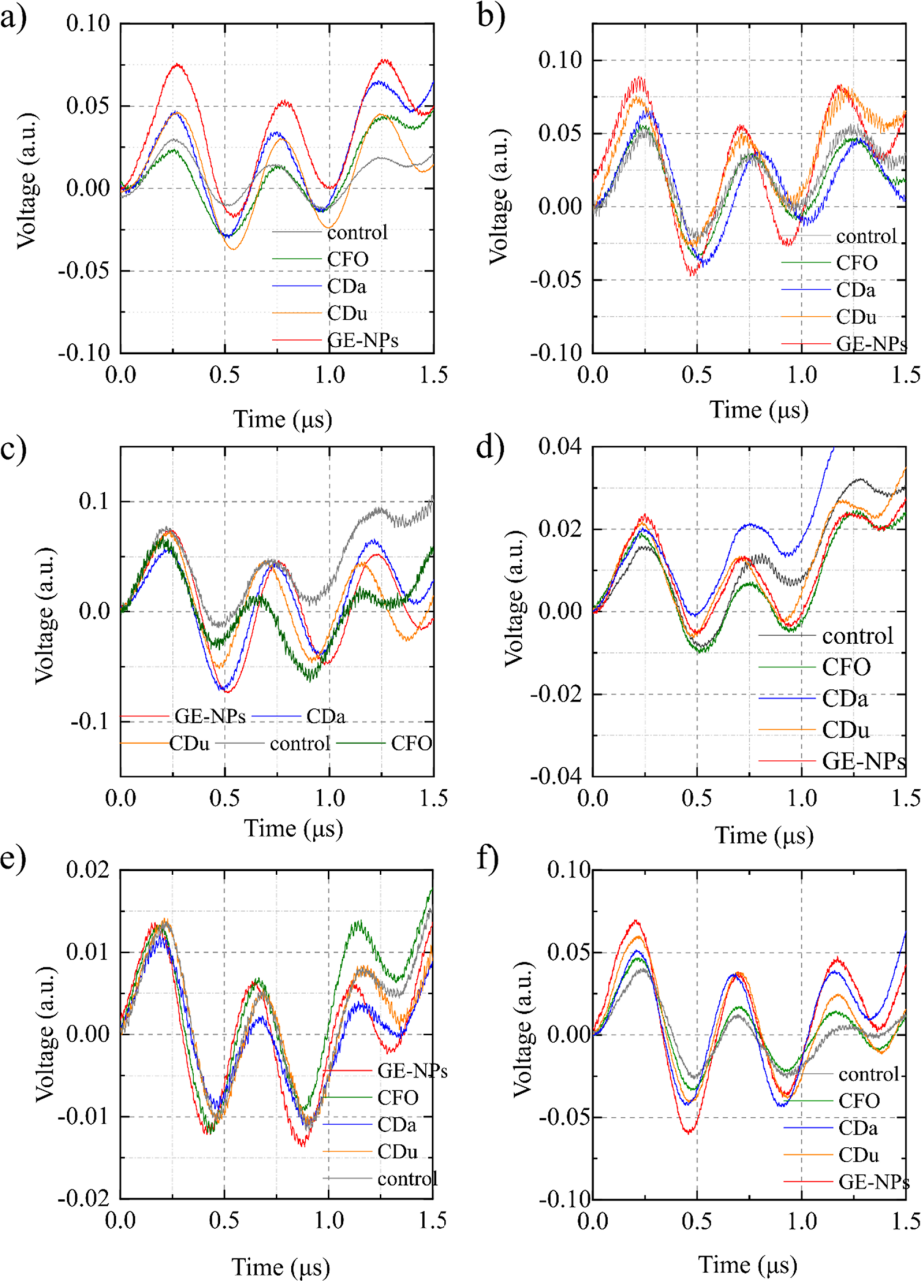
Photoacoustic responses recorded on **a** liver, **b** spleen, **c** heart, **d** gastrocnemius muscle samples, **e** lungs, **f** kidney. Con: control, CFO: carbon flurooxide nanoparticles; Cda: carbon nanodots from *β*-alanine; Cdu: carbon nanodots from urea-citric acid; GE-NPs: glucose-ethylenediamine nanoparticles

**Fig. 5:**
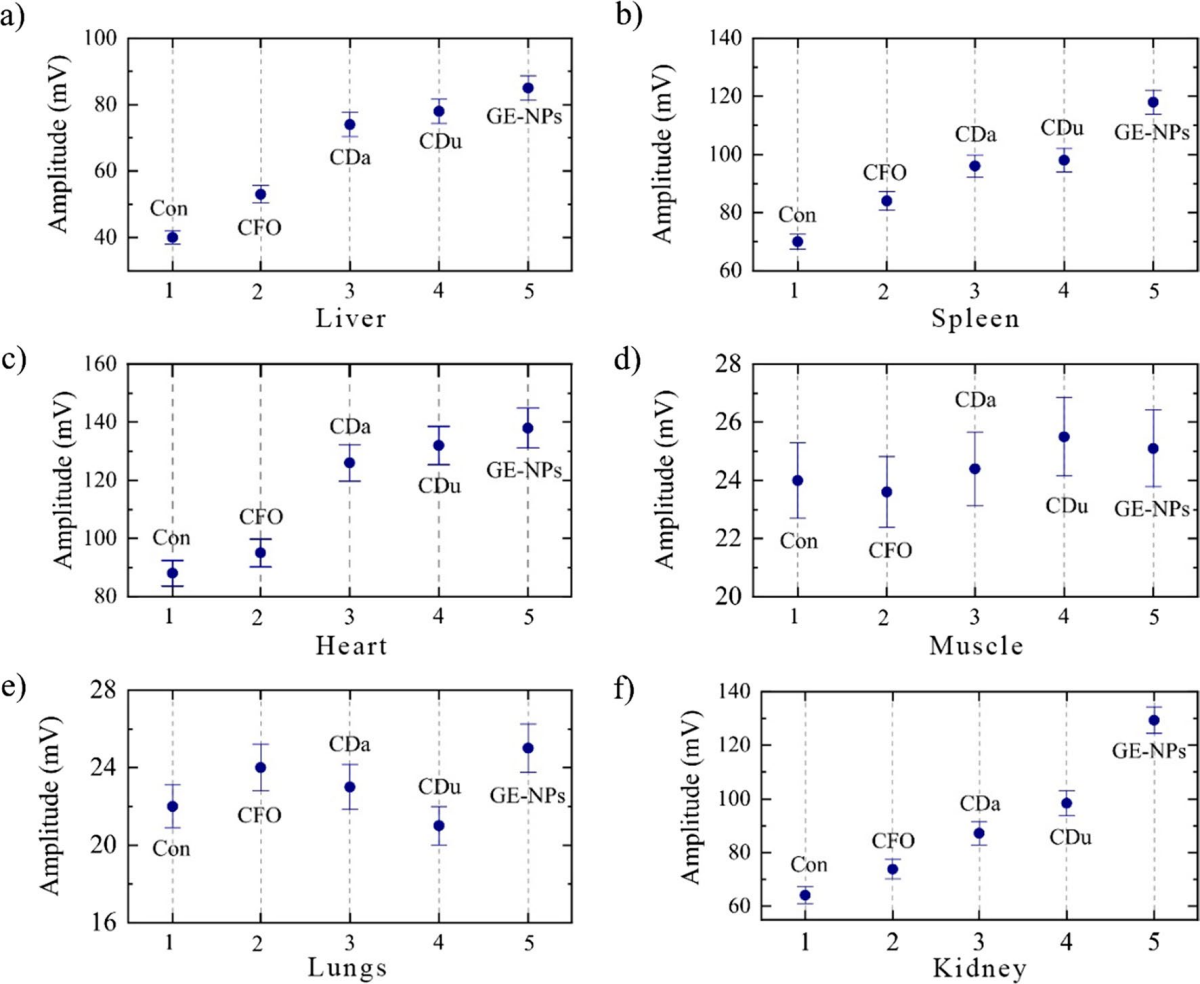
Averaged signal amplitude recorded on **a** liver, **b** spleen, **c** heart, **d** gastrocnemius muscle samples, **e** lungs, **f** kidney. Con: control, CFO: carbon flurooxide nanoparticles; CDa: carbon nanodots from *β*-alanine; CDu: carbon nanodots from urea-citric acid; GE-NPs: glucose-ethylenediamine nanoparticles

**Fig. 6:**
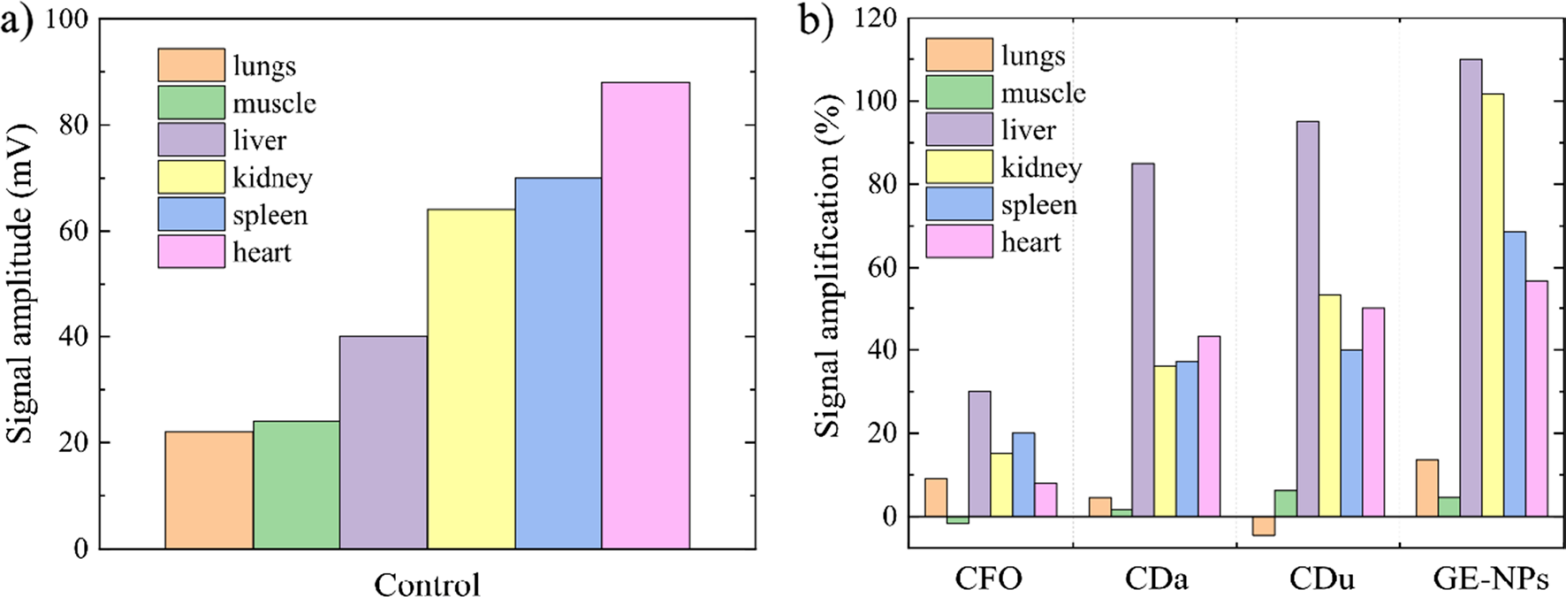
Comparative diagram of photoacoustic signals in different tissues. **a** Basal endogenous photoacoustic signal of the tissues without nanoparticles. **b** Photoacoustic signals induced by presence of different nanoparticles: CFO: carbon flurooxide nanoparticles; CDa: carbon nanodots from *β*-alanine; CDu: carbon nanodots from urea-citric acid; GE-NPs: glucose-ethylenediamine nanoparticles

As shown in Figs. 4, 5 and 6a, the intensity of background endogenous photoacoustic signals in control samples was small for all organs. In kidney, the maximal photoacoustic signal was obtained for glucose-ethylenediamine nanoparticles and minimal one for the carbon flurooxide nanoparticles. In the liver samples, the higher photoacoustic signals were recorded for glucose-ethylenediamine nanoparticles, while carbon nanodots from urea-citric acid and *β*-alanine demonstrated similar slightly smaller effects, contrary to the carbon flurooxide nanoparticles for which the corresponding photoacoustic signal was the lowest. In spleen samples, the higher photoacoustic signals were registered for glucose-ethylenediamine nanoparticles; whereas the carbon nanodots from urea-citric acid and *β*-alanine demonstrated similar effects. The signal from the CFO was very low. In heart samples, the signals generated by carbon flurooxide nanoparticles were very close to those on the control sample. Photoacoustic signal amplitude induced by presence of carbon nanodots from *β*-alanine, carbon nanodots from urea-citric acid, glucose-ethylenediamine nanoparticles had an upward trend. Quite strong natural heterogeneity of lungs did not allow to make a clear conclusion regarding nanoparticle accumulation in this tissue. No detectable photoacoustic signal enhancement due to the nanoparticles presence was found in all studied types of muscles, i.e. soleus, gastrocnemius, tibialis, extensor digitorum longus muscle.

Endogenous photoacoustic signals were more efficiently induced by presence of glucose-ethylenediamine nanoparticles (Fig. 6b, the fourth cohort of columns). Carbon nanodots from *β*-alanine and urea-citric acid sensitized very similar enhancement of photoacoustic signals in the organs as compared to the control samples (Fig. 6b, the second and third cohort of columns). Increase in the signal amplitude induced by carbon flurooxide nanoparticles was lower than that for other studied nanomaterials (Fig. 6b, the first cohort of columns).

## Discussion

This study demonstrated for the first time that photoacoustic signals can be significantly sensitized by presence of glucose-ethylenediamine nanoparticles, carbon nanodots from urea-citric acid and *β*-alanine, and to a lesser extent carbon flurooxide nanoparticles in liver, kidney, heart, spleen versus native background signals from endogenous tissue chromophores (e.g. hemoglobin). The photoacoustic signals were detected after intravenous administration of these carbon nanomaterials preliminary coated with anthological biocorona from the plasma proteins. Remarkable fluorescent properties of the carbon nanomaterials [22, 23, 28] make them multifunctional agents, which can be utilized for bi-modal fluorescence-photoacoustic imaging.

It is worth to note that the injected glucose-ethylenediamine nanoparticles have demonstrated the stronger photoacoustic response at concentrations 10 times lower than both carbon dots and carbon flurooxide nanoparticles. In this context, glucose-ethylenediamine nanoparticles can be recommended as perspective agents in photothermal imaging/therapy because they can effectively transform energy from absorbed light into heat and increase the temperature of the surrounding tissue.

Carbon nanodots as photoacoustic agents have recently attracted considerable attention due to their excellent physical and chemical properties [38]. For instance, carbon nanodots synthesized from native precursor material *Hypocrella bambusae* possess water solubility, broad absorption, red-light emission, low biotoxicity, and can highly generate singlet oxygen—^1^O_2_(0.38) and heat (27.6%) under 635 nm laser irradiation. These excellent properties of carbon nanodots synthesized from *Hypocrella bambusae* can be utilized for fluorescence/photoacoustic imaging-guided synergistic photodynamic and photothermal therapies [39]. Carbon nanodots synthesized using hydrothermal treatment of 1,3,6-trinitropyrene and Na_2_SO_3_ have the absorption range up to 1100 nm. They present strong fluorescence and generate ^1^O_2_ through two-photon excitation mechanism, and show photothermal conversion capability under irradiation by an 800 nm femtosecond pulsed laser. These features of carbon nanodots from 1,3,6-trinitropyrene/Na_2_SO_3_ together with good biocompatibility and broad absorption spectrum can be applied in photoacoustic and fluorescence imaging and photodynamic/photothermal synergistic cancer therapy using a single near infrared laser [40].

Our results are in accordance with the literature data regarding photoacoustic signal of carbon nanoparticles synthesized from food grade honey suspended with an organic macromolecular passivating agent ((*x*)-sorbitan mono-9-octadecenoate poly(oxy-1,2-ethanediyl) or PEG400), purged with argon and heated in a domestic microwave [41]. Carbon nanoparticles from honey were injected to mice intradermally and intravenously and it was revealed that liver was the dominating organ for nanoparticle accumulation when organs were collected at 2 h and 24 h post administration. In this context, the nanoparticle bio-distribution results in our study and the data of Wu et al. [41] are very similar. The other major organs for “honey” nanoparticle accumulation were kidney, lymph nodes and spleen. However, natural heterogeneity of the lungs did not allow to make clear conclusion on carbon nanomaterial accumulation in our study, whereas accumulation in the lung has been observed for both intradermal and intravenously injected carbon nanoparticles from honey [41].

The important finding of our study is the fact that photoacoustic signals of glucose-ethylenediamine nanoparticles, carbon nanodots from urea-citric acid and *β*-alanine, and carbon flurooxide nanoparticles can be registered after 24 h from their intravenous administration. It has been clarified that the carbon nanomaterials have long-term circulating time in the organism that makes them perspective prolonged circulating contrast agents in non-invasive photoacoustic imaging for acquisition pathologic information without radiation exposure.

Also, it can be suggested that longevity of staying of above carbon nanomaterials in the organism for 24 h allows them to be accumulated by tumor cells due to enhanced permeation and retention effect, i.e. passive nanoparticle targeting utilizing the pathophysiological properties of the tumor tissues, where preferential passive accumulation of nanoparticles is available due to the leaky and disorganized vasculature produced by that malignancies to sustain growth. Quantity and the sites of nanoparticle accumulation in tumor depend on their size and charge [16].

It should be also noted that the level of signal intensity decreased about four folds in the major organs of mice at 24 h time point post intravenous injection, as compared to 2 h time point in carbon nanoparticles from honey [41] that indicated the rapid clearance of the nanoparticles from the organism. After intradermal injection of “honey” nanoparticles to mice, the values of signals decreased approximately two times at 24 h time point as compared to 2 h time point indicating a slower mechanism of nanoparticle clearance [41]. In this context, limitation of our study is following, carbon nanomaterials were monitored in the organs of rats after 24 h from intravenous injection, and so we cannot conclude about dynamics of nanoparticle clearance from the organism.

In our previous studies, it was shown that carbon nanodots from *β*-alanine were much more toxic as compared to the nanodots synthesized from other organic precursors. Carbon nanodots from *β*-alanine increased the ambient level of excitatory neurotransmitter glutamate and inhibitory neurotransmitter GABA, decreased the initial rate of glutamate and GABA uptake and the exocytotic release of both neurotransmitters in rat brain nerve terminals [23, 28, 42, 43]. Interestingly, despite different toxic properties of carbon nanodots from *β*-alanine and urea-citric acid, both nanoparticles demonstrated almost similar biodistribution results shown in Figure 6b.

In perspectives, the photoacoustic signal registration using glucose-ethylenediamine nanoparticles can be applied as potential model and stand for detailed investigation of blood brain barrier permeability. For instance, increasing photoacoustic signal in the brain after administration of above particles can indicate increased blood brain barrier permeability during pathologies, e.g. inflammation, brain tumor, etc. or during medication or therapies. Long-term circulating time in the organism makes them perspective contrast agents in photoacoustic tomography, and favors their accumulation by tumor due to passive targeting and enhanced permeation and retention effect, and so cancer imaging. Due to high amplitude of photoacoustic signals and so high capability to transform energy from absorbed light into heat glucose-ethylenediamine nanoparticles can be recommended not only for photoacoustic imaging, but also for photothermal therapy of cancer.

## Conclusions

Summarizing, we report for the first time that photoacoustic signals of glucose-ethylenediamine nanoparticles, carbon nanodots from urea-citric acid and *β*-alanine, as well as carbon flurooxide nanoparticles can be registered 24 h after intravenous administration of these carbon nanomaterials in liver, kidney, heart, and spleen over native background signal provided by endogenous tissue chromophores. These carbon nanomaterials have long-term circulating time in the organism that makes them perspective as long-lasting contrast agents in photoacoustic tomography. Glucose-ethylenediamine nanoparticles more effectively transform energy from absorbed light into heat as compared to other studied nanomaterials that makes them perspective agents for photothermal therapy. Longevity of staying of the carbon nanomaterials in the organism for 24 h is expected to realize in their accumulation in tumor due to intensive passive targeting. Fluorescent properties of the above-mentioned carbon nanomaterials are making them multifunctional agents that also can be utilized in bi-modal fluorescence-photoacoustic imaging.

## Data Availability

Not applicable.
